# Utilization of Cerebrospinal Fluid Proteome Analysis in the Diagnosis of Meningioma: A Systematic Review

**DOI:** 10.7759/cureus.20707

**Published:** 2021-12-26

**Authors:** Rabia Choudhary, Adil Elabbas, Abhishek Vyas, Darin Osborne, Himaja Dutt Chigurupati, Lawahiz F Abbas, Prathima Kampa, Farzana M.H, Hooria Sarwar, Michael Alfonso

**Affiliations:** 1 Emergency Medicine, Internal Medicine, Neurology, California Institute of Behavioral Neurosciences & Psychology, Fairfield, USA; 2 Radiology, California Institute of Behavioral Neurosciences & Psychology, Fairfield, USA; 3 Family Medicine, California Institute of Behavioral Neurosciences & Psychology, Fairfield, USA; 4 Internal Medicine, California Institute of Behavioral Neurosciences & Psychology, Fairfield, USA; 5 Research, California Institute of Behavioral Neurosciences & Psychology, Fairfield, USA; 6 Psychiatry, California Institute of Behavioral Neurosciences & Psychology, Fairfield, USA; 7 Medicine, California Institute of Behavioral Neurosciences & Psychology, Fairfield, USA

**Keywords:** primary or brain metastasis, brain tumors, target protein biomarkers csf, micro rna, csf protein, cerebrospinal fluid (csf), serum biomarker, imaging biomarker, meningioma

## Abstract

Meningiomas have been classified as the most commonly occurring primary brain tumors. Although the majority of meningiomas are benign and slow-progressing, the tumors that grow to a larger size are associated with various risks during surgical procedures. Early detection of meningiomas is crucial to the treatment as those detected early can be treated through non-invasive methods. Due to their benign nature, meningiomas contain homogeneous protein biomarkers that can be easily identified. Cerebrospinal fluid (CSF) has a high protein composition which can be used to diagnose various brain tumors. Because CSF comes into direct contact with the brain during its functioning, it is one of the factors that makes it an important source of different biomarkers. An analysis of biochemical changes occurring in the CSF can be useful in assessing the condition of the periventricular white matter and the parenchyma.

In this review, PubMed, Medline, PubMed Central, and Google Scholar were used to identify studies discussing meningiomas regarding their assessment, types, diagnosis, and treatment, with more attention directed towards the application of CSF proteome analysis in diagnosis. Priority was given to studies published within the last 15 years. The following keywords were used in the literature search: “cerebrospinal fluid,” “meningiomas,” “brain tumors,” “primary brain tumors,” “protein biomarkers,” “proteome analysis,” and “diagnosis.” Subsequently, the 15 most relevant studies were selected for inclusion in the review. We excluded studies discussing different types of non-brain tumors as well as older articles. The selected studies also underwent a quality appraisal process using corresponding assessment tools. The selected articles were highly informative about meningiomas and the processes of diagnosis and treatment that are currently in use as well as those that are being developed or implemented. The use of CSF proteins in the diagnostic process is also discussed in this review. The studies also describe proteomics as a less invasive procedure that allows for the analysis of entire proteins and the projection of diagnostic images with higher resolutions that aid in the diagnosis.

## Introduction and background

According to the World Health Organization (WHO), meningiomas account for roughly 37.6% of all diagnosed primary brain tumors, and 50% of these are benign, implying that they are not harmful to the individual [1]. Meningiomas originate in the meningeal layers of the brain. They are generally categorized as grades 1, 2, and 3, which are also known as typical, atypical, and anaplastic meningiomas, respectively. The susceptibility and occurrence of meningiomas are linked to various factors in an individual’s life [1]. These factors include genetic abnormality, alcoholism, obesity, a family history of meningiomas, hormonal therapy, and exposure to various forms of radiation. Regarding their clinical manifestation, meningiomas present differently depending on the individual, and some individuals can be asymptomatic while others are symptomatic. Symptomatic individuals may present with abnormality in terms of the functioning of other body parts because of deficits in their neurological function [1,2].

Statistically, meningiomas are the most frequently occurring primary brain tumors, with incidence rates of 10.82 per 100,000 people annually and prevalence rates of 97.5 per 100,000 people annually [2]. Adults are also more likely to develop meningiomas, and the mean age of patients with meningiomas is 66 years [2]. On the other hand, the male-to-female ratio is 1:2, and the recurrence rates have been estimated to be approximately 20% within 10 years of removal, with higher rates observed in higher-grade meningiomas [2].

Meningiomas originate in the meningeal layers of the brain, and most are irregular, harmless, and slow-progressing tumors. They cause chromosomal mutations such as chromosomal deletions in the *neurofibromatosis type 2* gene [3]. Benign meningiomas mostly lead to a single or a few chromosomal deletions, while in malignant, rapidly progressing, and higher-grade meningiomas, several mutations have been observed to occur. While monosomy 22 is the most common genetic aberration detected in meningiomas, additional chromosomal abnormalities, signaling pathways, and growth factors have also been linked to its pathophysiology. The most common genetic abnormalities in early meningioma formation are losses on 22q12.2, a region encoding the tumor suppressor gene *merlin*. In malignant and high-grade meningiomas, alteration of chromosomes 9, 10, 14, and 18 has been observed, along with chromosome 17 amplification [4].

The diagnostic tool currently considered to be the gold standard is magnetic resonance imaging (MRI) of the brain [3]. Regarding the current treatment options, the approach depends on the benignity or size of the tumor, the clinical manifestation, as well as the speed at which it progresses. Benign tumors are treated through observation and frequent imaging, whereas meningiomas that are symptomatic, large, and rapidly progressing are treated surgically [3].

Recently, researchers have placed more focus on various cerebrospinal fluid (CSF) molecules that have the potential to be essential biomarkers in the diagnosis of various cancers that affect the central nervous system (CNS) [5]. In addition to the CSF coming into direct contact with the brain cells, other factors influence its suitability as a source of biomarkers, such as the fact that it is easily accessible and reduces the need for invasive methods of diagnosis. The CSF is can also reflect the actual pathology of the brain and the spine that form the CNS. CSF is secreted at the choroid plexus and its production and circulation in the body is a continuous process [5]. Direct contact with the brain occurs through the circulation process that also brings it close to the tumors, and therefore, some of the molecules in the tumor may be found in the CSF due to dispersion [5]. The availability of CSF proteins in large amounts contributes to their potential as biomarkers for various tumors including meningiomas [6]. Some of the proteins that have been used include human chorionic gonadotropin (HCG) and alpha-fetoprotein (α-FP). These components are obtained from various parts of the CNS through the use of lumbar punctures to collect samples [6].

In the use of these proteins in meningioma diagnosis, the meningioma-specific proteins in the CSF are first identified through analyses conducted using two-dimensional electrophoresis (2-DE) and mass spectrometry [6]. The results of these analyses are then verified via Western Blot analysis. This is done through the identification and observation of the various patterns that the different cell proteins express. Recent research has suggested the further use of these patterns to grade various meningiomas [6]. However, it is also important to note that even with the success attributed to the use of CSF proteins as diagnostic biomarkers, there is low popularity of these methods in the clinical setting, which begs the question: is the lack of research on the clinical feasibility of the CSF proteome analysis methods derailing quicker diagnosis of brain tumors? In this review, we discuss the applicability of this diagnostic approach in the clinical setting.

## Review

Methodology

This review follows the guidelines outlined by the Preferred Reporting Items for Systematic Reviews and Meta-Analyses (PRISMA) [7]. Data were sourced from Google Scholar and PubMed, and only peer-reviewed articles were included. Medical Subject Heading (MeSH) terms and keywords were employed to filter relevant articles about the utilization of CSF proteome analysis in the diagnosis of meningioma. The following keywords were employed in the literature search: “cerebrospinal fluid,” “meningiomas,” “brain tumors,” “primary brain tumors,” “protein biomarkers,” “proteome analysis,” and “diagnosis.”

MeSH Advanced Search

“Meningiomas” [Majr] OR Clear Cell Meningioma OR Xanthomatous Meningioma OR Meningiomas Xanthomatous OR Fibrous Meningioma OR Fibrous Meningiomas OR Intracranial Meningioma OR “Benign Meningioma” [Majr] OR Malignant Meningioma AND Cerebrospinal Fluid OR “cerebrospinal fluid proteins” [Mesh] AND “Tumor Biomarkers” [Mesh] OR Carcinogen Markers OR Biological Tumor Markers OR “Tumor Markers” [Mesh] OR Cancer Biomarkers.

Inclusion and Exclusion Criteria

The articles identified were filtered using the following inclusion criteria: priority was given to English-language articles published within the last 15 years. The papers were also prioritized based on the type of research carried out, with human brain cell experimental and observational studies given preference. Studies were also preferred based on the type of brain tumor investigated, with a priority given to those on meningiomas in line with the research objectives.

The exclusion criteria used to filter studies included in the review included studies involving other specific types of metastasized brain tumors and those that were descriptive in nature.

Quality Appraisal

The selected articles underwent quality appraisal to assess their quality using various existing tools. The Cochrane Risk of Bias assessment tool was employed to assess any bias in the studies transcending biases that could affect the overall quality of the clinical trials. The Newcastle Ottawa Scale was used to assess any bias in the observational studies used in the research. The Assessment of Multiple Systematic Reviews 2 tool was employed to assess the quality of systematic reviews. The Critical Appraisal Skills Randomized Controlled Trials Checklist was used to assess the quality of the results reported in the various studies used in the research.

Results

During the initial search for the articles related to meningioma and meningioma diagnosis, 188 articles were identified, which were then further filtered to 100 articles related to meningioma diagnosis through the use of CSF biomarkers, with 30 articles reporting the use of protein biomarkers. The selected articles underwent further screening to assess if they conformed to the inclusion and exclusion criteria, and a total of 15 articles were finally selected for inclusion in the review. The quality assessment did not reveal any quality concerns.

**Figure 1:**
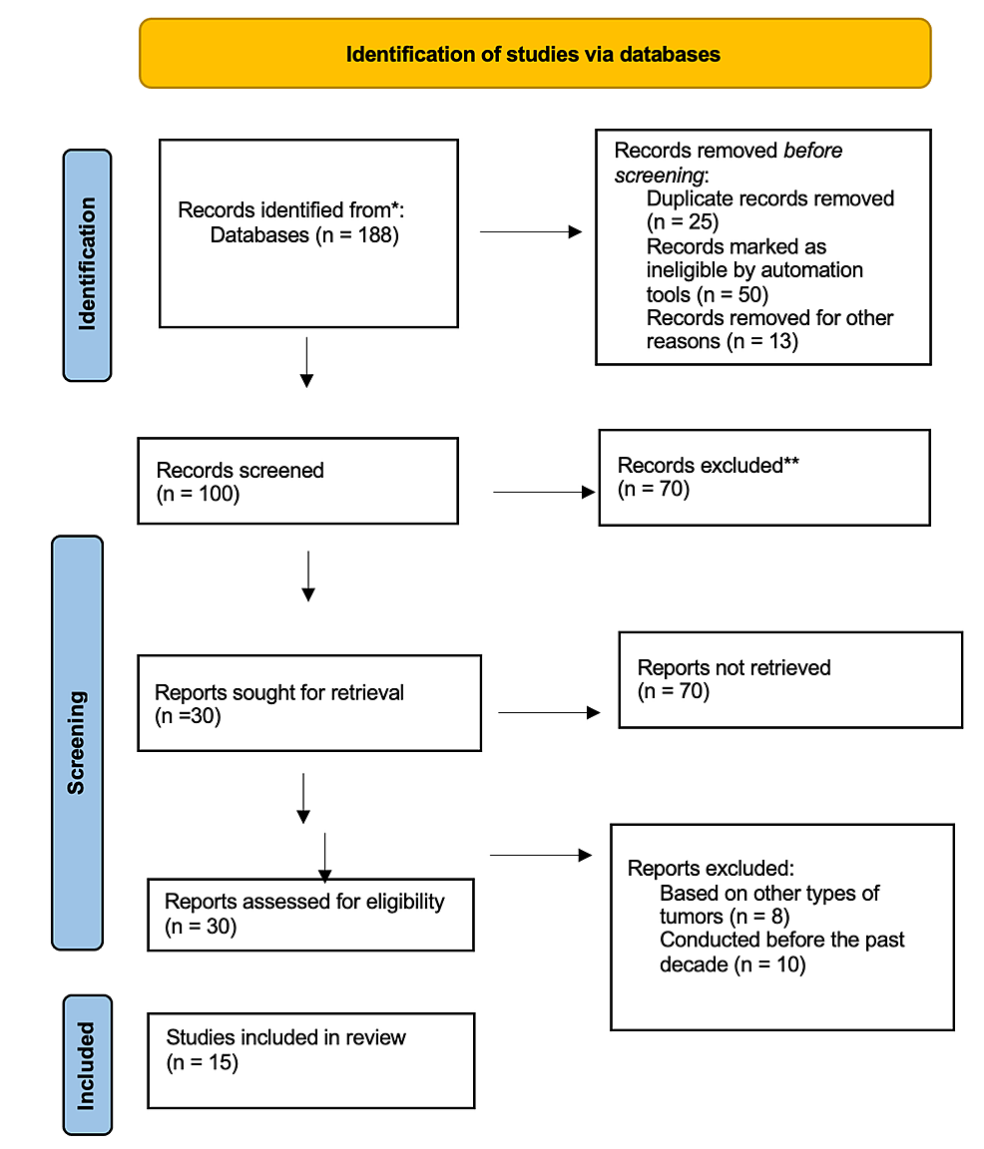
The Preferred Reporting Items for Systematic Reviews and Meta-Analyses flowchart.

Figure 1 illustrates the PRISMA flow chart of article identification displaying the many stages of the systematic review applied in the identification of studies.

Discussion

Current Methods and Diagnosis

The current methods employed for the diagnosis of meningiomas and other primary brain tumors are based on patients’ family history, physical examination, and as well as the use of radiological methods. Radiological methods include head contrast computed tomography (CT) scans and MRI. The concentration of water within living tissues is depicted by MRI, which is speculated to predict parts of their biomechanical activity. MRI signal intensity has been used to predict the consistency of a tumor and its histological subtype in meningiomas. Currently, MRI is the preferred gold standard of diagnosis [3].

Although effective, these methods have been reported to have various concerns, such as the invasive techniques currently in use, and this research has been conducted to find better methods, which has included numerous studies on various components that come into contact with the neural systems such as the CSF [8].The protein biomarkers in the CSF have been shown to aid in not only the diagnosis but also the treatment and assessment of recurring cancers, and therefore, can be used clinically by oncologists and other physicians [9]. The various components of CSF, particularly proteins, which are frequently found in higher concentrations, have been studied to determine their sensitivity in the diagnosis of brain tumors [10]. Consequently, success at the clinical level during the diagnosis of brain tumors can be accomplished, enabling research to be integrated into the practical setting [11,12]. The current methods also involve the use of diagnostic tools such as biopsies; however, the analysis of CSF proteins can lead to the identification of protein biomarkers which are obtained through less invasive methods such as a lumbar puncture [13]. The current methods of diagnosis may also lead to a late diagnosis, which worsens the prognosis, and it is therefore important to identify other biomarkers that increase the possibilities of an early diagnosis [14]. The identification of these proteins can also aid in the discovery of proteins that help track the progression of a brain tumor such as apolipoprotein E (APOE) [6].

Recently, research has been directed towards profiling Ki-67, an immunohistochemical marker that can correlate with severity and outcomes. The study by Liu et al. focused on pooled hazard ratios (HRs) for overall survival (OS) and disease/progression/recurrence-free survival (D/P/RFS) that were calculated using a fixed or random-effects model. The study comprised 43 trials with a total of 5,012 patients. In meningiomas, higher Ki-67 expression levels were linked to poor overall survival (HR = 1.565; 95% confidence interval [CI] = 1.217-2.013) and D/P/RFS (HR = 2.644; 95% CI = 2.264-3.087). They concluded that an increase in Ki-67 in patients with meningioma correlates with poor outcomes and an indefinite need of follow-up [15].

Most studies have emphasized that the early detection of brain tumors can lead to a better prognosis and it is important to implement ways that aid in early diagnosis [16].

Proteomic Analysis of Cerebrospinal Fluid

Proteomics technologies have become more sensitive and dependable because of recent improvements in mass spectrometry. Targeted proteomics, a relatively new field of mass spectrometry-based proteomics, has shown enormous promise in resolving the limitations of traditional molecular biology-based techniques such as Western blotting and immunohistochemistry [8].

Ghantasala et al. emphasized the necessity of focused proteomics in detecting and validating proteins involved in brain tumor pathophysiology. They demonstrated that APOE can be a potential tumor progression marker in meningioma based on the multiple reaction monitoring (MRM) tests utilizing CSF [8].

**Figure 2:**
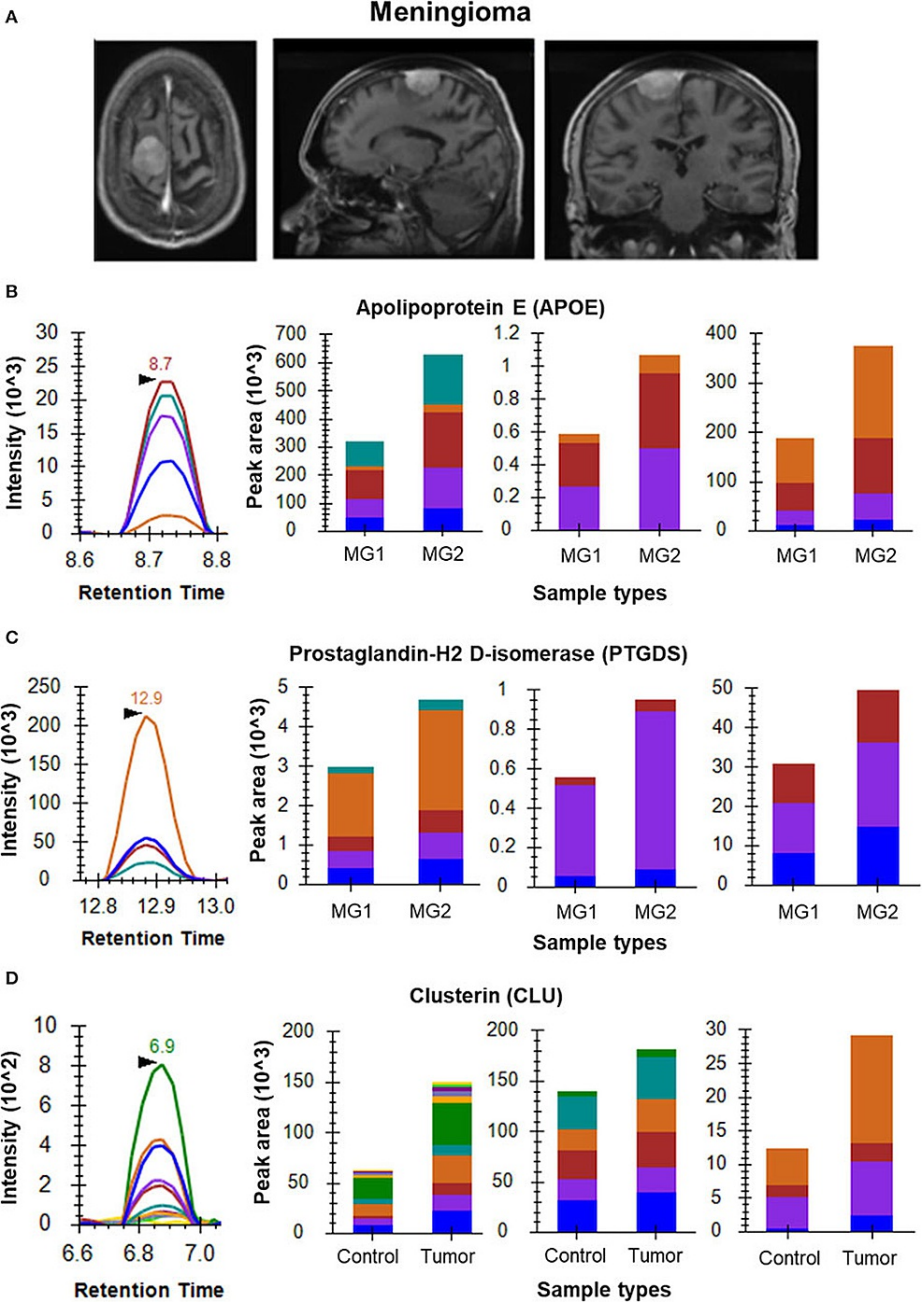
Multiple reaction monitoring approach. Copyright © 2021 Ghantasala, Pai, Biswas, Gahoi, Mukherjee, KP, Nissa, Srivastava, Epari, Shetty, Moiyadi and Srivastava. This is an open-access article distributed under the terms of the Creative Commons Attribution License (CC BY). The use, distribution or reproduction in other forums is permitted, provided the original author(s) and the copyright owner(s) are credited and that the original publication in this journal is cited, in accordance with accepted academic practice. No use, distribution or reproduction is permitted which does not comply with these terms.

Figure 2 depicts MRM used in the study by Ghantasala et al. The study showed how MRM, a targeted proteomics technique, was used to analyze peptides from proteins such as apolipoprotein A1 (APOA1), APOE, prostaglandin H2 D-isomerase (PTGDS), vitronectin, and complement C3 (C3) in CSF from meningioma patients [8].

Several factors are related to the effectiveness of proteome analysis as a method of diagnosing brain tumors, including the availability of the CSF proteins in large amounts, which increases the chance of interaction with the molecules produced by various brain tumors; the direct interaction of the CSF with the brain, as well as the nature of the proteins in terms of patterns. The proteome analysis methods are also less invasive as the sampling is done through a lumbar puncture [10].

CSF analysis can help with brain cancer detection and clinical prognosis. MicroRNA (miRNA) technology has rapidly enabled sensitive recognition of distinct miRNAs that are molecularly stable in the CSF and can distinguish between different kinds of CNS cancers. Analysis of CSF miRNAs can be a useful addition to clinical care due to the ease with which a CSF specimen can be acquired [10].

Because changes in CSF are believed to sensitively reflect pathological processes in the CNS, such as neoplastic disorders, it has been recognized as a valuable source for prospective biomarker screening in this era of proteomics [11]. Because of its great sensitivity and selectivity in detecting brain tumors, the integration of proteogenomics with targeted proteomic validation can be of crucial value in cancer research [8].

Meningioma Diagnosis

In one study, meningioma-specific proteins were identified through analysis of CSF samples using 2-DE and mass spectrometry. The results are then validated through a Western Blot analysis that analyzes the patterns and compares them to those of patients already diagnosed with meningiomas. The proteins identified include APOE, alpha-1-antitrypsin (AAT), and PTGDS proteins, as discussed above. The patterns on the proteins are also essential in grading meningiomas according to the grades discussed above [6].

**Figure 3:**
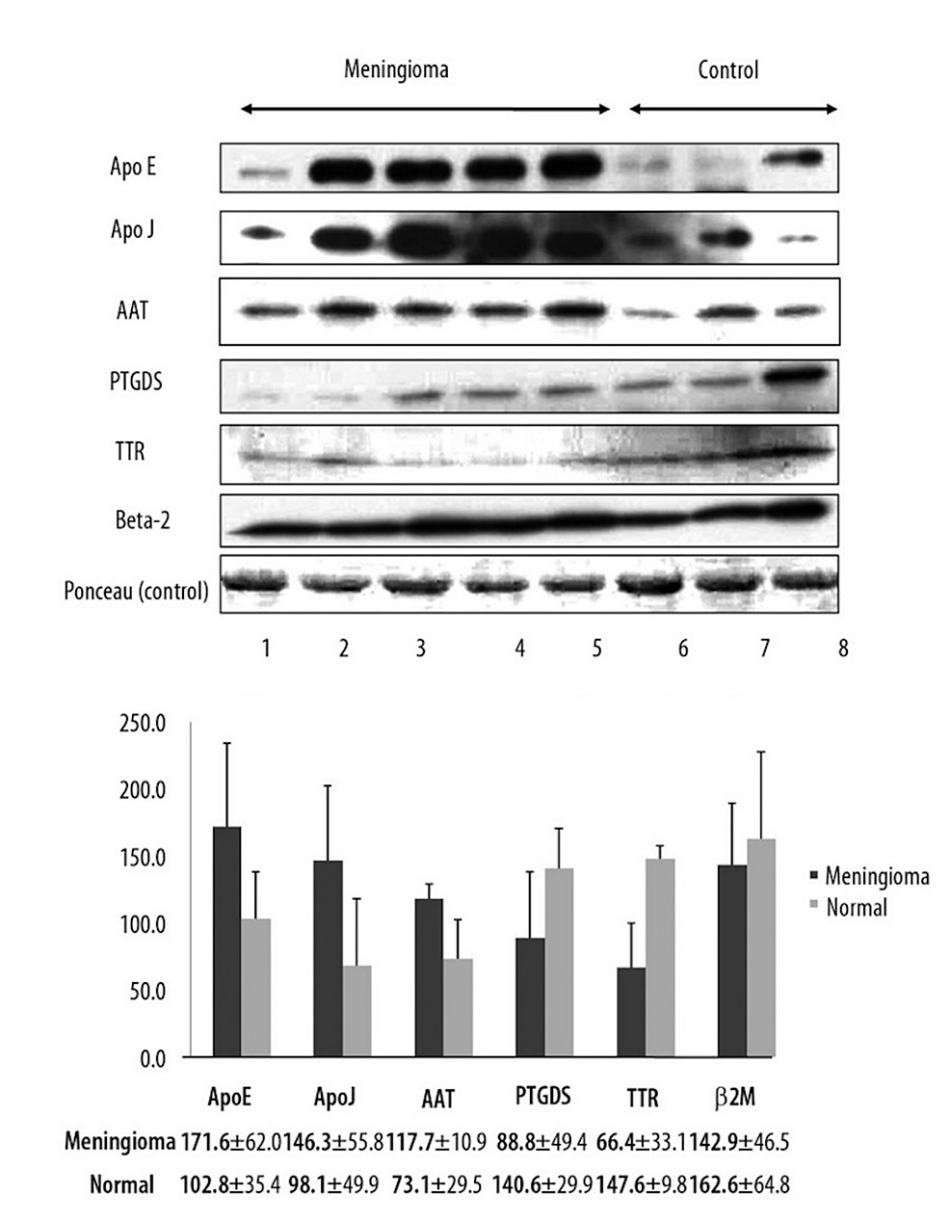
Western blot technique for the verification of additional CSF samples obtained from meningioma patients. CSF: cerebrospinal fluid Published under the Creative Common Attribution-NonCommercial-NoDerivatives 4.0 International (CC BY-NC-ND 4.0) allowing to download articles and share them with others as long as they credit the authors and the publisher, but without permission to change them in any way or use them commercially.

Figure 3 depicts the Western blot approach for the verification of additional CSF samples obtained from meningioma patients conducted in the study by Kim et al. [6].

Another study focuses on the use of miRNAs, which are proteins found in the CSF because it is also a readily available protein in the CSF that can aid in the early detection of brain tumors [14]. The PTGDS protein is also identified as a possible effective biomarker of brain tumors, with the current study vouching for its feasibility [17]. The studies that focus on existing literature discuss the perceived benefits of the use of proteomics and other CSF analysis in the diagnosis of various forms of brain tumors, including meningiomas, as well as reflect on their low popularity as a form of diagnosis. Therefore, more research is required on their clinical feasibility to aid physicians and oncologists in the field of diagnosing brain tumors in a faster and easier method that improves the eventual outcomes of the disease [14].

The study by Kopkova et al. showed how miRNA detection in CSF is a multi-step procedure that includes CSF management and storage, miRNA isolation and expression analysis, and final data standardization, analysis, and interpretation. The research emphasized that there is a clinical need for improved brain tumor biomarkers that would allow for more precise diagnosis, prognosis, and therapy response prediction. Brain cancers linked to miRNAs produced and identified in CSF appear to be promising proteins from this perspective. Unfortunately, several factors can influence the entire procedure, which are yet to be standardized [14].

Another study has defined CSF proteome analysis-based methods as those that are effective in the early detection of brain tumors. The effectiveness of early diagnosis leads to a better prognosis, which implies that the outcomes of most brain tumors can be improved after treatment before cancer progressively grows and metastasizes [16]. As already discussed, one of the advantages associated with the use of CSF in the diagnosis of meningiomas and other forms of brain tumors as a source of protein biomarkers is that it can easily be accessed through a lumbar puncture [10]. Thus, the use of CSF proteins is emphasized in the analyzed studies in the diagnosis of brain tumors as effective biomarkers [18].

MiRNAs are single-stranded, non-coding RNAs that are 18-25 nucleotides long and influence gene expression post-transcriptionally. These molecules are tissue-specific and play a role in the development of various neurological illnesses. Circulating miRNAs have been discovered in nearly all human bodily fluids, including CSF, and appear to be extremely stable and resistant to harsh circumstances. Furthermore, unregulated levels of CSF miRNAs have been linked to CNS tumors including meningiomas. Thus, miRNAs in CSF of brain cancer patients may potentially aid in the development of a novel diagnostic approach that allows for more accurate diagnostic techniques, including in patients with meningiomas [19].

The contribution of miRNAs to the clinical spectrum of benign meningiomas MiRNAs provide a second line of amplification of growth-promoting cellular signals by deregulating the translation of genes involved in signaling pathways known to be critical for meningioma genesis and progression. MiRNAs as indicators for meningioma diagnosis could be valuable and should be investigated further in the future [20].

The importance of miRNA as an important biomarker for meningioma is further solidified in the study by Slavik et al. It aimed to identify molecular genetic markers for more accurate prediction of meningioma recurrence and improved targeted therapy. The research included microarrays that revealed the presence of miRNA expression in primary and recurring meningiomas of all WHO grades. In a group of 172 patients, those reported to be unregulated were further verified using quantitative real-time polymerase chain reaction. The resulting dataset was statistically analyzed, and predictors of meningioma recurrence were discovered. The study concluded that MiR-15a-5p, miR-146a-5p, and miR-331-3p were found to be the most significant prognosticators in both adjusted and non-adjusted models of time to relapse. In several models, the final validation phase demonstrated the critical importance of miR-146a-5p and miR-331-3p, as well as clinical parameters such as the type of resection (total or partial) and WHO grade. Following stepwise selection in a multivariate model on an enlarged cohort, the model with decreased miR-331-3p expression (HR = 1.44; P = 0.001) and partial tumor resection (HR = 3.90; P = 0.001) was found to be the best predictive. Furthermore, in univariate models adjusted for clinical variables, both miRNAs remained prognosticators in the subgroup of total resections. Thus, the presented models may allow for a more accurate prediction of time to recurrence of meningioma and, consequently, optimum postoperative therapy. Furthermore, combining this model with current knowledge of the molecular pathways behind recurrence should allow for the identification of distinct meningioma subtypes and more precise therapy [21].

Shalaby et al. reported that the use of CSF analysis is not being implemented clinically for various reasons, including the lack of collaboration between oncologists and other physicians involved in brain tumor diagnosis and the researchers involved in carrying out this research. Therefore, a gap exists in the sharing of knowledge, thus delaying the transfer of this technology to the field where it would be most valuable. Shalaby et al. also note that the transfer of this technology would simplify the work done by health professionals and, as discussed above, aid in the early and effective diagnosis of brain tumors [5].

Application of Cerebrospinal Fluid biomarkers in Miscellaneous Conditions

CSF biomarkers play an important role in additional neurological condition in both the adult and pediatric populations.

Moyamoya disease (MDD) is a rare cerebrovascular disorder with an unknown cause, described by stenosis or blockage of bilateral internal carotid arteries and an abnormal vascular network. Araki et al. analyzed CSF samples taken from 20 patients with MMD and 12 healthy controls to discover biomarkers. Surface-enhanced laser mass spectrometry (SELDI-TOF-MS) with an anion exchange chip was used to generate mass spectral data in three distinct buffer conditions. A comparison analysis was performed after expression difference mapping, utilizing the derived protein profiles. The study concluded that the statistically significant biomarkers found in patients with MDD have the ability to detect and classify the disorder, implying that the CSF proteins may be important in the diagnosis of various brain disorders [18].

Multiple sclerosis (MS) is an autoimmune disease that affects the brain and spinal cord (CNS), leading to neurodegeneration. Biomarkers in body fluids may be able to predict and study neurological impairment in MS patients. CSF oligoclonal bands (OCB) are key indicators that can aid in MRI diagnosis and prevent false-positive diagnoses. New biomarkers such as aquaporin4 (AQP4) have already been established in clinical practice, while others such as anti-myelin oligodendrocyte glycoprotein (anti-MOG) and neurofilament light (NFL) are being investigated. When it is important to draw a distinction between a very active disease course and a mild progression, prognostic CSF proteome analysis may alter the treatment options for MS patients. Currently, the most promising prognostic CSF indicators include protein chitinase 3-like1 (CHI3L1) and NFL. Increased CSF concentrations of oligoclonal IgM bands (OCMB) and protein 14-3-3 have been linked to MS and a worsening of disease progression [22].

Huang et al. demonstrated the importance of T-cell activation in MS disease progression. Several chemokine receptor CXCR3 ligands, such as CXCL9/10/11, were shown to be overexpressed in the CSF of MS patients. This receptor is only expressed by Th1 cells and possibly certain regulatory T-cells. Researchers noted a higher infiltration of CCR5+ and CXCR3+ T-cells in active lesions, as well as higher production of the ligands MIP-1a and CXCL10. MIP-1a and CXCL10 levels in the CSF of MS patients are elevated. Several newly found CSF biomarkers, including CD6, IL7, and IL12B, as well as its component IL12A, have previously been related to inherited risk factors for various diseases. Many other CSF molecules, such as CHI3L1, IL-6, and CXCL13, have the potential to be used as clinical indicators; however, more research is needed to confirm their significance. The relevance of these proteins in MS is further supported by overlapping findings from proteomics and genetics [23].

Another important autoimmune neurological condition is autoimmune encephalitis (AE). It is an immune-mediated CNS disease that is not infectious. It causes immune-mediated brain injury which can manifest in various ways, ranging from mild cognitive impairment and behavioral abnormalities to life-threatening epileptic seizures.

Levraut et al. analyzed the CSF concentration of interleukins (ILs). In 20 AE patients, the researchers evaluated the CSF concentrations of each cytokine and compared them with ILs. Overall, 13 patients with CNS demyelinating disorders and 20 non-inflammatory controls had their IL-6 and IL-17A levels tested. The concentration of IL-17A in the CSF of AE patients was higher than in either of the control groups. The findings suggested that CSF IL-17A could be useful in determining the severity of AE at the outset. Consequently, CSF IL-17A could be a promising therapeutic target and an effective tool for evaluating early immunosuppressive therapy [24].

Neurodegenerative disorders are becoming more common, and new biomarkers for diagnosis, prognosis, and therapy efficacy are urgently needed. Alzheimer’s disease (AD) is a progressive neurodegenerative illness marked by cognitive deterioration and the presence of amyloid plaques and neurofibrillary tangles. It is the most prevalent cause of dementia, which is defined as a loss of cognitive (thinking, remembering, and reasoning) and behavioral abilities to the point where they interfere with everyday life and activities. The protein composition of the CSF reflects structural and functional brain changes. Kinney et al. highlighted different CSF proteins in the disease course; TNF, IL-6, and TGF are involved in inflammation, disease progression, and neurological decline [25]. In retrospect, CSF levels of NFL protein, a marker of neuroaxonal degeneration, are associated with cognitive impairment in patients with AD and frontotemporal dementia [26].

Primary brain tumors are the most prevalent solid tumors and the main cause of cancer-related death in children under the age of 18 [13]. Improvements in protein identification and quantification methodologies, as well as through proteome investigations and the newly emerging area of proteogenomics, are directed at enhancing our understanding of the molecular pathophysiology of pediatric brain cancers [12].

The efficiency of brain tumor treatment is dependent on early tumor diagnosis, thus it is critical to identify and implement biomarkers that can help. One such biomarker is miRNAs, which have been studied but require further research to optimize their role as biomarkers [14].

**Figure 4:**
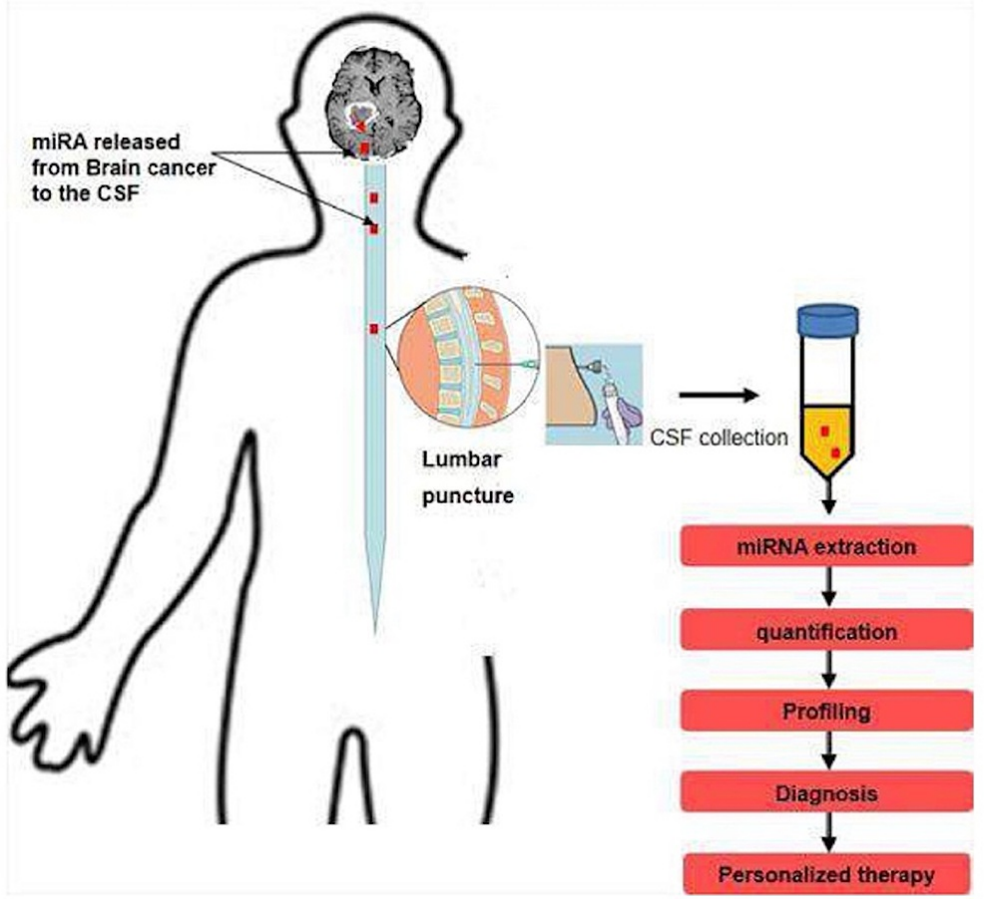
Cancer releases proteins into the CSF that can be extracted and used to diagnose meningiomas by profiling and histological identification. CSF: cerebrospinal fluid Published under the Creative Common Attribution-NonCommercial-NoDerivatives 4.0 International (CC BY-NC-ND 4.0) allowing to download articles and share them with others as long as they credit the authors and the publisher, but without permission to change them in any way or use them commercially.

Figure 4 illustrates how cancer releases proteins that are circulated in the CSF, which can be extracted and used for diagnosis by profiling and histopathological identification of meningiomas [10].

**Figure 5:**
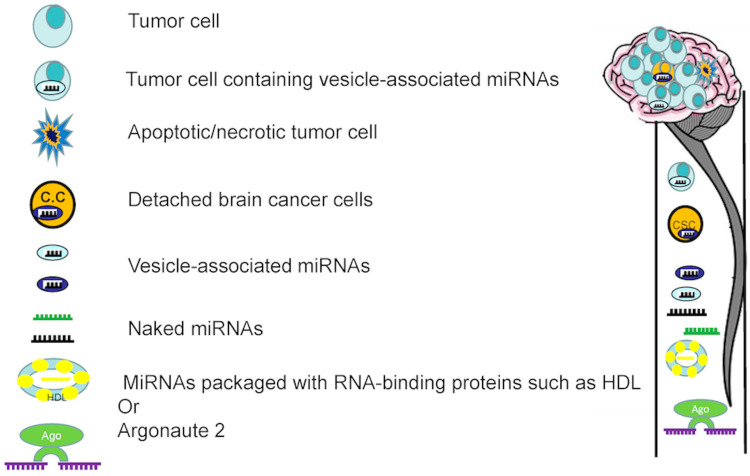
Illustration of putative miRNA origins and types in the CSF. miRNA: micro RNA; CSF: cerebrospinal fluid Published under the Creative Common Attribution-NonCommercial-NoDerivatives 4.0 International (CC BY-NC-ND 4.0) allowing to download articles and share them with others as long as they credit the authors and the publisher, but without permission to change them in any way or use them commercially.

Figure 5 shows putative miRNA origins and types in the CSF. Brain tumor-associated miRNAs in the CSF can be actively released into the CSF by brain tumor cells in the form of smaller membrane microvesicles (e.g., exosomes) or RNA-binding proteins (e.g., HDL or Argonaute, Ago). Another concept is that dead or dying cancer cells release miRNAs into the CSF, where they remain intact and complexed to Ago outside of the cell. Additionally, bare miRNAs (those lacking exosomes or microvesicles) may be generated actively by brain tumor cells or passively released by apoptotic or necrotic cells. A third hypothesis is that a subset of miRNAs is linked to CSF-circulating detachable brain cancer cells [10].

Therefore, protein biomarkers in the CSF can be useful in not only diagnosing but also treating and monitoring reoccurring tumors, which can help oncologists and other physicians clinically, both in the pediatric and adult populations [8].

Bruschi et al. compared CSF samples obtained from the extraventricular drainage (EVD) of 17 non-tumor individuals with CSF samples sourced from the EVD of 29 consecutive patients treated for common brain tumor types, encompassing both malignant and benign histopathologies, and discovered some possible protein biomarkers. The goal of the study was to use the large volume of CSF produced by EVD to look for predictive protein biomarkers for assessing the likelihood of a cancerous condition versus any other requiring an EVD, despite the presence of abnormal CSF production (controls). The study concluded that potential biomarkers could distinguish between different types of brain tumors to some extent [27].

Apart from the miscellaneous conditions discussed above, CSF protein analysis may play a fundamental role in pediatric neuropsychiatric diseases such as autism. Because brain-specific proteins are not found in other tissues, non-invasive blood testing can be used to detect structurally and functionally damaged brain areas, as well as the severity and prognosis of neuropsychiatric disorders [28]. For example, low levels of neonatal CSF vasopressin correlated with a late diagnosis of autism in kids [29].

To conclude the application of CSF protein in miscellaneous conditions, CSF protein evaluation plays an important role in more current events such as the coronavirus disease 2019 (COVID-19). In the study by Tandon et al., CSF immunoglobulin M (IgM) and a combination of severe acute respiratory syndrome coronavirus 2 serum and CSF reverse transcriptase-polymerase chain reaction (CSF RT-PCR) testing were explored in patients with COVID-19 who had neurological symptoms. CSF RT-PCR, CSF (IgM) tests, and CNS-targeting antibodies can be used together to guide diagnosis, treatment, and outcomes. The most prevalent finding of CSF analysis was an increase in CSF protein, which was occasionally accompanied by an increase in mainly lymphocytic cell count. CSF protein levels were shown to be increased in all stages of neurological disease [30]. Other studies have shown biomarkers of CSF, such as NFL protein and glial fibrillary acidic protein, correlate with brain injury in patients with COVID-19 [31].

Overall, treatment for cancer is increasingly being tailored based on tumor-specific genetic changes in the modern era of medicine. Next-generation sequencing (NGS) of tumor-derived circulating nucleic acids in CSF can help with precision treatment in the field of brain cancer. Baumgarten et al. examined if NGS from the CSF of neuro-oncologic patients could reliably detect tumor-specific genetic alterations and if it could assist clinicians in better managing patients with CNS cancer. In 23 of 27 patients, NGS proved successful (85%). In 20 out of 27 patients, 74% had genomic changes, and 11 (40%) were potentially actionable. Therefore, the study found that NGS from CSF fluid is practical in clinical practice and delivers therapeutically significant changes in a wide sample of patients. This method could be integrated into a cancer medicine program to enable patients with brain tumors to have more therapeutic alternatives [32].

Table 1 lists the tabulated analysis of selected studies that were found to be relevant to meningiomas and the diagnosis and treatment techniques that are currently in use as well as those that are still being developed and implemented. The use of CSF proteins in diagnostic processes is also discussed. Proteomics, according to the studies, is a less invasive method that allows for the examination of entire proteins and positions this technology as an addition to the current diagnostic tools.

**Table 1:** Tabulated analysis of the selected studies. CSF: cerebrospinal fluid; EVD: extraventricular drainage; APOE: apolipoprotein E; PTGDS: prostaglandin D2 synthase; miRNA: micro RNA; PCR: polymerase chain reaction; GAL: galanin; HSPA5: heat shock 70 kDa protein 5; WNT4: wingless-type MMTV integration site family, member 4; CNS: central nervous system; MMD: moyamoya disease

Author/Year	Purpose	Type of study	Subjects	Results	Outcomes
Bruschi et al. (2021) [27]	To understand the potential biomarkers of childhood brain tumor identified by proteomics of CSF from EVD	Systematic review	CSF from EVD from 29 children bearing different brain tumors and 17 controls needing EVD insertion for unrelated causes	1,598 and 1,526 proteins were identified by liquid chromatography-coupled tandem mass spectrometry proteomics in CSF control and brain tumor patients, respectively; 263 and 191 proteins were exclusive of either condition	The method was able to differentiate between brain tumor vs. non-tumor/hemorrhagic conditions (controls) and to differentiate between two large classes of brain tumors
Ghantasala et al. (2021) [8]	To study the importance of targeted proteomics in the detection and validation of proteins with roles in pathobiology of brain tumors	Randomized controlled trial	17 CSF Samples	In the CSF samples of patients with meningioma, the peptides of APOE proteins resulted in a cumulative fold change of 2.21 while the peptides of PTGDS proteins resulted in a fold change of 1.52	A study of the CSF proteins could be used in place of surgery in the diagnosis of meningioma
Slavik et al. (2020) [21]	To indefinity meningioma patients at high risk of tumor recurrence using miRNA profiling	Cohort study	In a cohort of 172 patients, deregulated miRNA was further confirmed using quantitative real-time polymerase chain reaction	The most important prognosticators were miR-15a-5p, miR-146a-5p, and miR-331-3p, according to the adjusted and non-adjusted models of time to relapse. miRNAs were still prognosticators in meningiomas	The presented models may allow for more accurate prediction of time to recurrence of meningioma
Xiao et al. (2020) [9]	To describe and properly discuss the clinical roles of distinct classes of CSF biomarkers, isolated from patients with brain tumors	Systematic review	Various studies on the role of CSF in brain tumors	CSF proteins such as miRNAs and circulating tumor DNA among others can be used to detect and assess the progression of brain tumors as a form of liquid biopsy	The protein biomarkers in the CSF can be essential in not only the diagnosis but also the treating procedures, as well as in assessing recurring tumors, and thus aid the oncologists and other physicians clinically
Kopkova et al. (2019) [19]	CSF miRNA signatures as diagnostic biomarkers in brain tumors	Prospective monocentric study	175 patients with brain tumor and 40 patients without tumors but have hydrocephalous as a control experiment	5 miRNA levels were successfully identified and upon CSF diagnostic scores for various brain tumors, and the specific tumors were defined and differentiated from healthy samples	This was the largest research on miRNA and brain tumor diagnosis and it concluded that the protein biomarker can be useful not only in the diagnosis but also the prognosis of brain tumors
Kopkova et al. (2018) [14]	To highlight the potential of CSF miRNAs as diagnostic, prognostic. and predictive biomarkers in brain tumors and to summarize technological approaches for detection of CSF miRNAs	Systematic review	Multiple CSF samples taken of patients with various neurological disorders including brain tumors	MiRNAs are often components of circulating fluids including CSF and have been found to be prospective diagnostic biomarkers for patients with brain tumors including meningioma	The effectiveness of treatment for brain tumor patients depends on the early detection of the tumor, and it is therefore important that various biomarkers that can lead to this be identified and implemented. This includes miRNAs which have been under research and require more exploration to optimize their use as biomarkers
Shalaby et al. (2016) [10]	To discuss the potential and limitations of CSF analyses in brain cancer patients	Systematic review	Various types of CSF analysis employed in the diagnosis of brain tumors	The various CSF components such as various proteins and miRNA show potential in the field of oncology but there is still a long way to go to bring this to the clinical setting	CSF could provide critical biomarkers for use in the oncology field, especially in the diagnosis of CNS conditions. However, for the benefits of this technology, it is essential research and collaboration between researchers and clinical practitioners
Ludwig et al. (2015) [20]	To discuss post-transcriptional deregulation of signaling pathways in meningioma subtypes by differential expression of miRNAs	Systematic review	Compared the expression of 1,205 miRNAs in different meningioma grades and histological subtypes using microarrays and independently validated deregulation of selected miRNAs with quantitative real-time PCR	Identified 13 miRNAs deregulated between different subtypes of benign meningiomas, and 52 miRNAs deregulated in anaplastic meningioma compared with benign meningiomas	MiRNAs contributes to the clinical spectrum of benign meningiomas
Shen et al. (2014) [11]	To review the current available knowledge on the use of CSF proteome analysis as diagnostic biomarkers for brain tumors	Systematic review	Current published research on the use of CSF proteome analysis in the diagnosis of brain tumors	Various proteins such as GAL, HSPA5, and WNT4 were observed to be in lower quantity in patients with brain tumors	The various proteins identified in the study could bring about clinical breakthrough in the diagnosis of various forms of brain tumors
Anagnostopoulos and Tsangaris (2014) [12]	To summarize the current knowledge on research applying proteomics techniques or proteomic-based approaches performed on pediatric brain tumors	Systematic review	Currently available knowledge on research that applied proteome analysis methods in the diagnosis of brain tumors	Various proteins such as calcyphosin among others that can be potential biomarkers in the diagnosis of brain tumors were identified and discussed	Proteomics provide hope for a better future in the field of oncology in the diagnosis of brain tumors
Samuel et al. (2014) [13]	To analyze the existing literature on proteomics in oncology of the neural systems and their contribution to the clinical practice	Systematic review	All current available literature on proteome analysis in oncology	There are various biomarkers that can be used in the diagnosis of various brain tumors as well as other conditions of the CNS, and these biomarkers can be obtained from CSF analysis	The analysis of CSF for biomarkers could provide diagnostic tools for various brain tumors and conditions at the clinical level and help move away from the current invasive methods such as biopsies
Kim et al. (2012) [6]	To identify meningioma-specific CSF proteins from 4 patients with a meningioma and 4 patients with a non-brain tumorous lesion	Randomized controlled trial	Four patients with meningioma and 4 patients with a non-brain tumorous lesion	The proteins APOE, apolipoprotein J and alpha-1-proteinase inhibitor were increased while the proteins PTGDS, transthyretin, and β2M were decreased	APOE could be a critical protein biomarker when tracking how meningiomas progress in the brain of an individual
Weston et al. (2011) [16]	To understand CSF analytic methods that improve the sensitivity of the diagnosis of brain cancer	Systematic review	Methods currently under research in CSF analysis	There are various methods such as flow cytometry, PCR, and analysis of other biomarkers that can be applied in tumor diagnosis	The effectiveness of early diagnosis implies a better prognosis, and CSF analysis methods lead to the early detection of brain tumors
Rajagopal et al. (2011) [17]	To demonstrate the feasibility of centrally collecting and processing high-quality CSF samples for proteomic studies within a multi-center consortium	Randomized controlled study	33 children with medulloblastoma and 25 age-matched control subjects.	PTGDS was discovered to be of lower amount in brain tumor samples by six-fold when compared to the control samples.	PTGDS can be an important biomarker of brain tumors
Araki et al. (2010) [18]	To identify novel biomarker candidate proteins differentially expressed in the CSF of patients with MMD using proteomic analysis	Randomized controlled study	20 persons suffering from MMD and 12 control subjects	34 protein biomarkers were identified in the CSF of MMD patients	The identified biomarkers have the capability to diagnose and classify MMD, which indicates that CSF proteins can be essential in diagnosis of brain conditions

The use of a highly sensitive modality in the diagnosis of brain tumors, particularly meningioma, has been an important topic that many researchers, including the aforementioned researchers, are aiming to answer. The use of CSF protein analysis to diagnose meningioma early is one that merits further attention because it allows the diagnosis of asymptomatic meningioma patients, potentially giving patients a better prognosis.

The current work focuses on a sensitive technique for diagnosing brain meningioma that enables early detection of the disease. As a result, the management can be changed. Although it is not extensively used by clinicians, it might be of utmost significance in the future.

Limitations

Each type of study covered has its own set of limitations. The number of samples collected for analysis in studies that use CSF directly may be less than what is required for accurate and dependable results. However, this might be solved by using replication to obtain more samples, as shown in the study by Kim et al. The variety of studies employed in research primarily focused on studying literature may be limited, resulting in a limited amount of knowledge about the topic. Further, the majority of the studies included in this study concentrated on meningiomas and other types of primary brain tumors, leaving secondary brain tumors, which are also common. Another limitation is the limitation to articles that have been published in the last 15 years. Because many of the studies were based on previous research, this could result in a limited knowledge base as there may be older researches with results that could contribute significantly to the study.

## Conclusions

In conclusion, brain tumors are rampant in the population and their prognosis depends on early diagnosis which influences the treatment. The current diagnostic methods for brain tumors include the use of CT scans and MRI, with the latter being considered more effective. However, these diagnostic methods have been described as invasive, either with the use of contrast dye or related to other invasive procedures. The use of circulatory fluids such as CSF that come into direct contact with the brain to detect brain tumors has been under investigation for years, with promising results for future easier, faster, and less invasive techniques of diagnosing brain tumors. Meningiomas are a type of brain tumor that are classified into typical, atypical, and anaplastic types. CSF has been recently identified as a reliable source of protein biomarkers for brain tumors because it has a high protein content and comes into direct contact with the brain.

Despite the favorable recommendations, the implementation of these procedures in the clinical setting has not attained the expected popularity. This is due to a lack of communication between researchers and oncologists and clinicians who interact with patients directly. There is also ongoing research on the application of this type of analysis to other types of brain tumors. Consequently, CSF proteomic analysis can be used as a diagnostic tool for brain tumors, which would be a significant contribution to health care by improving current diagnostic methods, which are invasive and time-consuming, and by increasing the likelihood of early detection of brain tumors, which would improve the prognosis and the quality of life for brain tumor patients.
